# Equitable health: let’s stick together as we address global discrimination, prejudice and stigma

**DOI:** 10.1186/s13690-018-0291-3

**Published:** 2018-08-22

**Authors:** Julie Babyar

**Affiliations:** Vallejo, USA

**Keywords:** Health equity, Discrimination, Prejudice, Healthcare bias, Health disparities

## Abstract

Tackling discrimination permanently in healthcare is not insurmountable. It is achievable. Discrimination is costly in lives, in healthcare delivery and waste, in human capital, in financial resource and even in healthcare improvement initiatives that do not adequately account for its impact. Healthcare must understand the underlying inequalities each faces from the start and tailor care toward equal health outcomes. Solutions have been offered and should be funded and evaluated. Additionally, a global plan to address discrimination and bias in healthcare must be consistent, accountable and be shaped around standardized tools and measures. The idea that an individual is better or more important than another has no place in today’s world, particularly in health. Therefore, it is critical that each is provided his or her individual needs to achieve best outcomes. It is critical for healthcare to advance health equity. Global healthcare must do its part to be a team leader on this issue.

## Introduction

Everyone should be provided equal care and an equal opportunity to realize full health potential. The majority realize this truth, yet remain unaware of the depths of inequality and discrimination in healthcare. Too, the problem of discrimination in healthcare becomes so monumental and multidirectional that it often seems insurmountable. Tackling discrimination permanently in healthcare is not insurmountable. It is achievable. In fact, equality in health opportunity is achievable through health equity. Equality in health innovation and workplace opportunity is also achievable, and should be addressed within the same scope.

Direct and indirect discrimination in healthcare is studied and measured utilizing several distinct definitions. Health equity, equality and healthcare disparities are main foci in discrimination and bias research. The Robert Wood Johnson Foundation (RWJF) describes health equity as “the ethical and human rights principle that motivates us to eliminate health disparities, which are differences in health or its key determinants (such as education, safe housing, and freedom from discrimination) that adversely affect marginalized or excluded groups. Disparities in health and in the key determinants of health are the metric for assessing progress toward health equity.” [1]. The achievement of health equity is a worldwide goal. Health equality refers to treating all the same, abiding by the definition of equality as the state of being equal [2]. It is crucial to understand the difference in equality and equity. The RWJF believes health equity to be both process and outcome [1], whereas other health entities believe equity to be the process, a means to provide individuals the outcome of health equality [3]. Regardless of process or outcome view, health equity has become a collective focus in public health worldwide.

## Background

It is not enough to treat everyone equal in healthcare. Healthcare must understand the underlying inequalities each faces from the start and tailor care toward equal health outcomes. In order to achieve this, health disparities must be identified and targeted. The U.S. National Library of Medicine defines healthcare disparities as “the variation in rates of disease occurrence and disabilities between socioeconomic and/or geographically defined population groups” [4]. Taking it a step further, most health associations, governance and policy analyses incorporate specific vulnerable groups during the assessment of disparities. Common groups analyzed in health disparities include those in specific ethnicity and race classes, gender classes, socioeconomic and income class and those of minority sexual preference classes. Health disparity research has grown stronger after the Institute of Medicine’s 2003 report on discrimination in healthcare. Since this report, health service research has focused on stereotyping, prejudice and discrimination in clinical encounters as well as how perceived discrimination affects a person’s reception and willingness to receive medical care [5]. It is important to understand and differentiate between health equality, health equity and discrimination.

Racial and ethnic discrimination, ageism, gender bias, prejudice against sexual minorities and disability discrimination are all present in health. Too, discrimination is seen in all levels of healthcare. Much current literature centers on discrimination of patient care in health delivery. Literature even demonstrates that those with public insurance feel more discriminated against by their providers [6]. Major reported barriers and hurdles in healthcare operations are rooted in prejudice as well. These operations include but are not limited to healthcare administration, academic research leadership, clinical research selection and healthcare interpersonal business interplay.

The World Health Organization (WHO) and United Nations (UN) have recently highlighted discrimination in health delivery, workforce practice, national laws and overarching policies as areas in need of healthcare improvement. Much of this discrimination is seen through the denial or improper treatment toward specific groups in the provision of healthcare services, gender based workforce and economic norms, and national policies that do not adequately address human rights [7].

In the United States, multiple health organizations monitor and report on health disparities, including the American Public Health Association (APHA), the National Association of County and City Health Officials (NACCHO), the Department of Health and Human Services (DHHS) and many of its subsidiaries, including the Agency for Healthcare Research and Quality (AHRQ). Many of these agencies as well as nonprofit partners report on the state of health equity and health disparities, using national benchmarks in quality and reported chronic condition outcomes. The AHRQ report is a detailed, comprehensive analysis that retrieves data from 45 databases. In 2012, the AHRQ reported that black and Hispanic Americans received worse care than whites in 40% of quality measures, persons of American Indian and Alaskan Native descent received worse care than whites in 33% of quality measures and Asians received worse care than whites for 42% of measures. Notably, of each of these race categories, only Asians received better care than whites for a similar percentage as their receiving of worse care. In this report, it was also found that the lesbian, gay, bisexual and transgender (LGBT) population was less likely to report getting prescriptions filled in a timely manner, less likely to report getting access to non-urgent care and less likely to report that is was easy to see a specialist. A major barrier to care for the LGBT population is same sex insurance barriers. The report also showed a stark contrast in access to care between those in poverty and those not. Individuals with complex activity limitations had worse care for 18 measures than individuals without basic or complex activity limitations. Individuals with basic activity limitations also had worse care for 18 measures compared to those without basic or complex activity limitations. Finally, compared to urban populations, rural populations are less likely to receive preventive care and see their provider less often. This statistic is alarming because rural residents are more likely to be in poor health with more chronic conditions than their urban counterparts [8]. Additionally, people with disabilities, compared to those without disabilities, report foregoing healthcare due to cost 2.5 times more than their counterparts. They are 1.5 times more likely to be the victim of a nonfatal violent crime, have 3–4 times higher risk of cardiovascular disease, report higher rates of obesity, smoking and are at higher risk of injury. Those with cognitive disabilities are 5 times more likely to have diabetes compared to the general population [9].

The healthcare workforce in the United States is offered some protection from discrimination in the form of federal law. Workforces in other countries are not as fortunate, and WHO distinctly highlights female healthcare workers as susceptible to pay discrimination, physical violence, sexual violence and inability to participate in decision making and healthcare leadership. Within the United States, minority participation in executive healthcare leadership was reported at 11% in 2015, and minority participation in first and mid level office managers is at 19%, despite 32% of the patient population identifying as minority. In academic medicine, differences in minority representation remain stark. The Association of American Medical Colleges monitors underrepresented in medicine population, which consists of racial and ethnics populations not represented in medicine proportionate to their numbers in the general population. A 2010 report noted that while African Americans, Hispanics and Native Americans comprise 25% of the U.S. population, minorities only represent 7.5% of students in predominantly white medical schools [10]. Too, US medical schools have been recently examined for reasonable accommodation assistance for disabled students, with most not showing provision of reasonable accommodation in compliance with ADA laws [11]. Women now represent 21% of medical faculty in the United States [12]. Even in research study design and analytics, there is a lack of sex and gender equality in sample data [13]. This has become a major concern and focus for clinical research in recent years, as gender differences affect treatment efficacy, diagnostics and other research impacts. Finally, research funding gaps and disparities are found and perceived as discriminatory. In 2006, it was reported that despite almost three times as prevalent as cystic fibrosis, sickle cell disease research received one third of the grant funding [14]. Notably, the WHO and UN reports do not capture discrimination data on race and ethnicity as detailed as the United States.

### Current considerations

It is difficult to capture true and full scope of discrimination and health disparity in any one group due to many factors, including social determinants. Social determinants such as food access and home environments are often indirect forms of social prejudice that factor into health. Additionally, each country has its own cultural prejudices, discriminatory policies and intricate health system issues to tackle. Capturing comprehensive, accurate data on discrimination, prejudice and stereotyping in healthcare, from patient to administration workforce, is not currently available. The current process in the United States, recent global spotlight on the issue as well as other countries’ research initiatives all have potential in the creation of this comprehensive future.

Additionally, discrimination may not even be measured through standard classifications such as race or ethnicity. Without accounting for country or indigenous region of origin, a discrimination research study looked at a large sample of Latin Americans and found that basic categories of skin color produced striking differences in self reported health. The darker the skin color, the poorer the self reported health [15]. It is crucial to understand the dynamics, both broad and basic, that discrimination and perceived discrimination touches.

Discrimination and bias are also found in patient education as well as patient work. In 2006, a study indicated that most sex workers have never revealed their work to their medical provider, which is a serious individual and public health risk [16]. Nine years later this mistrust and hesitancy among sex workers persisted. In a 2015 study that determined sex workers do not have transparency nor trust with their medical providers [17]. Providers may tailor conversations and wording based on an individual’s work or education achievements, displaying either negative or positive discriminatory practice. However, healthcare workers may not even be aware of patient perception of discrimination.

Much discrimination and patient perception goes un- or underreported. An underreported area in healthcare prejudice is that of ageism. One in 5 adults over the age of fifty has experienced discrimination [18].

Discrimination and stigma in healthcare has significant, serious effects. In fact, individual and societal impact of discrimination in healthcare disrupts the primary oath in medicine to first do no harm. Discrimination in healthcare is so harmful, however, that it has become a major focus in research. Prejudice in healthcare negatively and disproportionately impacts stroke, cardiovascular, obesity, diabetes, hypertension, depression and anxiety among those discriminated against [19]. The perception of discrimination also impacts satisfaction, a major focus in current healthcare. Patient satisfaction is associated with better adherence, improved symptoms and overall better health outcomes. If discrimination impacts satisfaction, it therefore impacts outcomes, adherence and symptoms [19]. Discrimination in the United States contributes to adverse health outcomes and increased mortality in specifics populations. Overall, health disparities have been associated with $60 billion in excess costs as recently as 2009 [20].

The UN has acknowledged that discrimination affects both patients as well as health care providers [7]. In fact, it has been widely recognized that an under-addressed issue on the topic of discrimination is the negative effect it has on all. It is argued that this consideration is important not just to detail any misunderstood perceived discrimination, but to also compare and highlight major differences in racial discrimination. In fact, all forms of bias, discrimination and prejudice are crucial to understand and report on.

Perceived discrimination may or may not be able to be supported in actual metrics. In example, studies have found those with sickle cell disease perceive discrimination based on their disease, no necessarily race [21]. Other research has found that those with sickle cell disease wait 25% longer, explained in part by African American race disparity. This same research found that, even accounting for race and triage, those with sickle cell disease wait 50% with a long bone fracture. [22]. Perceived discrimination arguably adds to poorer health and psychological stress, regardless of measured discrimination, and all forms of discrimination and its effects must be accounted for.

Psychological and psychosocial research on prejudice clearly demonstrates impact in health, just as there is evidence of physical harm due to discrimination in healthcare [5]. In a study incorporating European countries in which societies have differing acceptance of lesbian, gay and bisexual individuals (LGB), LGB individuals reported better self rated health and wellbeing when accepted [23]. Additionally, allostatic load continues to be researched and considered in social study. The allostatic load over a woman’s lifetime, the accumulation of chronic stressors, has been examined from the standpoint of discrimination and prejudice, and this is impactful in health.

There is contradicting evidence among racial discrimination of black American women and health outcomes. In one study, actual and perceived discrimination was associated with adverse birth outcomes but not hypertension [24]. Another literature review revealed that there are mixed results on associations between racial discrimination and adverse birth outcomes [25]. Importantly, literature that does not provide associations between discrimination and health outcomes must not be interpreted as a complete and final word, nor does it encompass all health. In example, while some studies may not have shown that discriminated women of minority race deliver babies with lower birth weight, the studies do not detail the effect that their higher levels of depression have on their own bodies [25]. Too, allostatic load is not factored into much research.

Racial discrimination and feeling discriminated against significantly impacts scores of healthcare quality in the United States [26]. Thus, even in quality metrics, discrimination becomes a factor that is not easily stratified in everyday healthcare delivery survey. Discrimination is costly in lives, in healthcare delivery and waste, in human capital and even in healthcare improvement initiatives that do not adequately account for its impact.

Discrimination in the workforce affects the entire public, from wage gap economic impact to life quality. Medical underrepresentation affects hospital and healthcare operations, medical innovation, culture competence, community trust and research priorities. Research that is funded based on private interest and lobby may not be representative of population priority, nor is there much discussion on integrating and partnering in research for multi-outcome benefit.

Discrimination based on diagnoses also affects one’s quality of life. As efforts to achieve health equity target economic, housing and education, they must also continue diligence within healthcare. Physical and psychological wellbeing are impacted by direct and indirect daily prejudice. While eliminating prejudice and discrimination is a global effort, driven from individual, local and regional culture change, healthcare must do its part.

### Strategies to combat discrimination in healthcare and achieve equity

Addressing discrimination in healthcare is unlike any other healthcare issue in need of problem-solution resolution. The basis for discrimination is complex and solutions are heavily reliant on culture and behavior change. Additionally, leadership requires long term, sustained, consistent and dogged vigilance in keeping this issue at the forefront. In times of political agenda, during emergencies and health crises, among latest concerns on acute topics such as an emerging infectious disease and alongside continued popular priorities such as rare disease, health equity is an issue threatened to be sidelined. WHO, in conjunction with the UN, has begun to formally address discrimination in healthcare. In effort to achieve 2030 Sustainable Development Goals, the UN has detailed serious and significant prejudice in healthcare [7]. These efforts must remain priorities.

Strategies to reduce or eliminate prejudice and health disparity are abundant in literature. Instead of reinventing the wheel, it is prudent to follow guidance. Instead of allocating more resource to continued pocketed evidence, it is prudent to allocate resource to the strategies outlined. To manage discrimination and prejudice in healthcare, there must be cohesive, structured, comprehensive management of the issue at all levels. In example, civil rights and more recent anti-discrimination laws written into legislation such as the Affordable Care Act have created a sound foundation for this management. Since 2010, it has been formally illegal in the United States for healthcare institutions to discriminate based on race, color, national origin, sex, age, or disability in certain health programs or activities. This law is enforced through reporting access and tied to funding of government services.

A primary strategy to address discrimination and advance health equity lies within interdisciplinary research. The foundation for successful management toward healthcare equality and health equity requires interdisciplinary collaboration. Social and cultural academic, philosophical and policy expertise must weigh in to create effective resolution in healthcare discrimination. Over the past several decades, this interdisciplinary collaboration has taken shape. Policies and actions may be well intentioned, and may not even be originally interpreted as having an impact on health equity or discrimination. Interdisciplinary teams assist in analyzing and communicating need for change effectively. Additionally, research design and implementation continues to evolve with the assistance of interdisciplinary teams.

High quality research is a must in current future studies. Measurement tools in research have been reviewed, critiqued and studied for reliability and validity. The Everyday Discrimination Scale (EDS) has been adapted for use in healthcare studies, a prime example of interdisciplinary effort in discrimination work. The EDS has recently been assessed for reliability and validity in multiple studies, showing positive and demonstrable consistency, reliability and validity in black [27], American Indian and Alaskan native populations [28] as well as for Asian, Caucasian and Hispanic/Latino populations, with limitations to a few item differences [27, 29]. Several other tools, such as Detroit Area Study Discrimination Questionnaire (DAS–DQ), have also been studied for validity and reliability [30]. Overall, stronger studies for comprehensive discrimination measures that capture accurate, valid and reliable data among many stigmatized groups would be of benefit. Still, a foundation initiating from racial discrimination studies through use of a widely accepted tool, the EDS, can expand for gender, age, disability and sexual minority analysis.

An additional strategy requires that plans include immediate and continuous public health monitoring, ongoing data collection, interventions tailored to best practice, and continuous evaluation research of highest quality. Even definitions, terminology and categories like racial and ethnic groups are inconsistent. Ongoing health oversight provides for consistency and a means to standardization in terminology. Additionally, current evaluation research, education, programs and surveillance are widespread and seemingly dispersed without ability to capture. Instead, continuous reports would provide diligent and determined action. As example, to achieve parity for oral health in older adults, a Medicaid report for California was written detailing a systems approach [31]. This report could be included in comprehensive state to national analysis, on an annual basis, utilizing best evidence and current year data.

Another strategic move is to require healthcare to be a team player in everyday discrimination confrontation, through individual, regional and global achievement in elimination of bias of healthcare diagnoses. Some examples include basic clinical education on the transmission of HIV/AIDS to healthcare workers, policy expertise on emergent communicable diseases such as Ebola, removal of healthcare bias against those with mental illness and even remote campaigns utilizing targeted approaches to integrate strong health messaging and health interventions for children with albinism in Africa [32]. Importantly, scientific base and clinical medicine may drive change. In Ethiopia, for example, gains in perceived acceptance of those with HIV have been realized after status disclosure [33]. In contrast, campaigns for HIV treatment are proven less effective when countries do not first tackle stigma and discrimination [34]. Strategies that are proven effective should be afforded resources to buildout. Strategies to address discrimination should also be part of medical and clinical education, as well as ongoing quality metrics. It is recommended that those with disabilities, vulnerable to discrimination, be included as healthcare partners in their care [35]. Management and education of non-clinical healthcare providers is also an important step toward reducing discrimination in healthcare [19]. This is not only true for racial discrimination, it is true for bias against diagnoses as well. Mental illness and addiction are negatively prejudiced against by the public, and those with mental illness and homeless have also reported discrimination in healthcare [36].

The current state of discrimination, including perceived discrimination,should be a part of continuous public health at every level. It is important that local agencies have a clear picture of prejudice in healthcare not only for awareness, but also to assess, address and intervene locally when able. In example, 12.5% of Hawaiians reported refusal or poor care by a provider due to sexual orientation. 3% of persons in one county delayed mental or physical care due to stigma, while 19% delayed care in another county. Analyzing these trends as part of annual public health reports can assist in direct, immediate interventions between neighboring communities [37].

Patient voice must be included as a strategy to address discrimination and promote health equity. In example, patients with autism have suggested provider education, wording change, lighting and environmental changes in clinical locations [38]. Basic needs such as these can be met with ease.

Strategies that include patient voice in targeted health interventions are found for indigenous populations as well. Again, instead of reinventing the wheel or continuing to consume continuous literature on outcomes of health discrimination against indigenous populations, it is crucial that nations follow sound, produced strategies. The data and analyses provided through annual public health reports up to global health bodies can provide ongoing evaluation. In example, strategies to achieve health equity in Canadian indigenous populations have been produced in literature as well as through national delivery [39]. Not only should the information and evaluation be completed at request, it should be provided as an expectation. Successes may even be mimicked, with tailoring, for other nations seeking guidance. Instead of reinventing the wheel, maximize resource and follow already produced direction.

Strategies may also include basic provider and patient partnerships in cross cultural education. Often, immigrant populations face health disparities due to cultural and linguistic barriers. Efforts to increase provider cross cultural education, the provision of materials in native language and access internet education with translation capabilities are all strategies that help to achieve health equity [40]. Efforts that promote cross cultural understanding may assist in perceived witnessed and actual discrimination in health delivery as well.

Discrimination itself, perceived or actual, is a significant source of stress, anxiety and may lead to many adverse and negative health outcomes. Mental health is adversely affected by discrimination, even in those who have experienced discrimination due to their prior criminal convictions [41]. Discrimination is not discussed as part of an internal medicine or primary care provider question, but it should be. This would help in healthcare delivery, appropriate referral and diagnosis as well as open discussion on quality of life. Additionally, this would help in medical research and healthcare quality analytics because of the accuracy of data.

Because total and complete elimination of discrimination in any society is not yet apparent, mediation and mitigation strategies should be studied. One journal highlights the effect that good sleep quality has on reduction of perceived discrimination. Sleep quality and duration were found to account for 15–25% of the adverse effects of perceived discrimination. Improving sleep quality may be a strategy to mitigate the effects of perceived discrimination [42].

Mediation strategies may also include education at the public level on discrimination, bias, prejudice and coping with encounters. While unacceptable at every level, discrimination is prevalent, and arrives at various levels according to the prejudiced individuals’ perception. In dealing with this reality, and the reality that no two people have the exact same prejudices nor display them the same, it may be of benefit to review mediation strategies. It may also be of benefit to learn and maximize interpersonal relations that guide change, to the public as a whole. Mitigation and mediation may not prevent the sting, but they can work to soothe it until effective change comes.

Medical education and provider accountability are key strategies to reduce and eliminate discrimination in healthcare. As years of rotation increase, medical students have been shown to demonstrate more unacceptable mocking and disdain for specific groups of patients, such as the obese, mentally ill and difficult patients. While one study had limitations, the qualitative data surrounding it is profound and completely unacceptable [43]. That this behavior, modeled by select physicians, is allowed is an example for urgent and immediate change in healthcare delivery. Implicit racial bias has even been named as an issue that must be addressed in oncology training [44]. Cultural change at this level can be driven by policy, should be initiated in management and academic leadership, but must be measured in quality. Quality metrics must include measures on discrimination and professional bias in healthcare. What is and is not acceptable in healthcare is modeled bidirectionally. It should not just be modeled based on workplace discrimination law, it must be modeled by rewarding positive culture and designed by raising open and welcoming medical professionals. Medical education should continue efforts to provide a more statically representative population for all minorities and for all populations assessed in health disparities.

Perhaps the best strategy to achieve health equity and address discrimination is to ensure it is assessed and analyzed from local to national to global reports. Member states of the UN should provide detailed analyses in standardized templates and tools. Definitions and understanding of prejudice and discrimination must be detailed, exact and real. Though cultures, communities and biases differ vastly around the globe, the concept remains the same: the idea that an individual is better or more important than another has no place in today’s world. Healthcare must do its part to be a team player, even a team leader, on this issue. Templates, tools and reports should be standardized and checked for accuracy across the globe. Objective, trustworthy organizations like Human Rights Watch can provide valuable information. Discrimination, prejudice and stigma are often grown from culture but accepted through various countries’ governance [34]. Global cooperation in health processes and outcomes can hold communities accountable as well as provide for a means to achieve cultural acceptance. The WHO and UN must take care to be detailed and explicit in data, from every country, that incorporate race and ethnicity. The best strategy forward is by global cooperation, standardized measures and methodology, annual reports and continued global plans.

Healthcare operations and management must continue to assess collected demographics and begin interpreting health disparities for patients. Healthcare management associations must also continue accountability work in striving toward minority representation in management, operations and decision making. As successful models continue, other communities, countries and regions should be provided advisory assistance. Different cultures may require tailoring of rollout, and successful operations are more likely with mentorship and peer support. Indeed, there is much continued racism and prejudice throughout the world [45]. This is identified and addressed globally. To effectively participate in academia, healthcare operations or healthcare business on an international level, the global collective must be determined to provide for inclusive approach. Even at the business table or business association conference on another continent, healthcare must remain diligent and unwavering in commitment to equality in the workforce as well as equal voice in leadership and decision making, regardless of gender, disability or race. This can only be achieved with stronger global commitment to workplace policy as well as business commitment to inclusive leadership.

## Conclusion

Equality in health is often viewed as simultaneously idealistic and existent. In fact, health equality is neither. All populations, governed communities and individuals must continue striving toward equality in health. It is prudent to address current inequalities in healthcare, understand health equality and health equity terminology and work toward achievable parity. Tackling discrimination in healthcare must be accomplished for health service delivery as well as in healthcare leadership prejudice. In making these moves, individuals and collective society can acknowledge that we are not where we want to be, but it is not idealistic to strive toward total equality through health equity and determination.

## Abbreviations

AHRQ: Agency for Healthcare Research and Quality
APHA: American Public Health Association
DAS–DQ: Detroit Area Study Discrimination Questionnaire
DHHS: Department of Health and Human Services
EDS: Everyday Discrimination Scale
LGBT: Lesbian, gay, bisexual and transgender
NACCHO: National Association of County and City Health Officials
RWJF: Robert Wood Johnson Foundation
UN: United Nations
WHO: World Health Organization
